# Grey Matter Microstructural Integrity Alterations in Blepharospasm Are Partially Reversed by Botulinum Neurotoxin Therapy

**DOI:** 10.1371/journal.pone.0168652

**Published:** 2016-12-16

**Authors:** Hanganu Alexandru, Muthuraman Muthuraman, Venkata Chaitanya Chirumamilla, Nabin Koirala, Burcu Paktas, Günther Deuschl, Kirsten E. Zeuner, Sergiu Groppa

**Affiliations:** 1 Department of Neurology, University of Kiel, Kiel, Germany; 2 Movement Disorders and Neurostimulation, Department of Neurology, Neuroimage Center (NIC) of the Focus Program Translational Neuroscience (FTN), University Medical Center of the Johannes Gutenberg-University Mainz, Mainz, Germany; University of Zurich, SWITZERLAND

## Abstract

**Objective:**

Benign Essential Blepharospasm (BEB) and hemifacial spasm (HFS) are the most common hyperkinetic movement disorders of facial muscles. Although similar in clinical presentation different pathophysiological mechanisms are assumed. Botulinum Neurotoxin (BoNT) is a standard evidence-based treatment for both conditions. In this study we aimed to assess grey matter microstructural differences between these two groups of patients and compared them with healthy controls. In patients we furthermore tracked the longitudinal morphometric changes associated with BoNT therapy. We hypothesized microstructural differences between the groups at the time point of maximum symptoms representation and distinct longitudinal grey matter dynamics with symptom improvement.

**Methods:**

Cross-sectional and longitudinal analyses of 3T 3D-T1 MRI images from BEB, HFS patients prior to and one month after BoNT therapy and from a group of age and sex matched healthy controls. Cortical thickness as extracted from Freesurfer was assessed as parameter of microstructural integrity.

**Results:**

BoNT therapy markedly improved motor symptoms in patients with BEB and HFS. Significant differences of grey matter integrity have been found between the two patients groups. The BEB group showed lower cortical thickness at baseline in the frontal-rostral, supramarginal and temporal regions compared to patients with HFS. In this group BoNT treatment was associated with a cortical thinning in the primary motor cortex and the pre-supplementary motor area (pre-SMA). Contrary patients with HFS showed no longitudinal CT changes. A decreased cortical thickness was attested bilaterally in the temporal poles and in the right superior frontal region in BEB patients in comparison to HC. Patients in the HFS group presented a decreased CT in the left lingual gyrus and temporal pole.

**Conclusions:**

Although patients with BEB and HFS present clinically with involuntary movements of facial muscles, they exhibited differences in cortical thickness. While BoNT therapy was equally effective in both groups, widespread changes of cortical morphology occurred only in BEB patients. We demonstrated specific disease- and therapy-dependent structural changes induced by BoNT in the studied hyperkinetic conditions.

## Introduction

Benign Essential Blepharospasm (BEB) and Hemifacial Spasm (HFS) are the most common movement disorders affecting the face. BEB is a bilateral condition characterized involuntary closure of the eyelids caused by spasms of the orbicularis oculi muscle[1]. In contrast, HFS is characterized by unilateral, intermittent muscular contractions of the eye or facial muscles [2]. Both conditions are clinically similar, but BEB is considered to result from a dysfunction of the basal-ganglia-cortical loops with an involvement of the sensorimotor cortical regions [3, 4], while muscular contractions in HFS are caused often by vessel compression or irritation of the facial nerve [5]. Botulinum neurotoxin (BoNT) therapy is the standard symptomatic treatment for both conditions [6].

The exact pathophysiological changes leading to BEB are not completely clear. Sensory discrimination is disturbed in BEB patients [7, 8] and processing of tactile stimuli is impaired due to abnormal sensorimotor integration [9]. In patients with BEB grey matter increases in the bilateral putamen were detected in a structural neuroimaging study [10]. However, this finding was contrasted by another voxel based morphometry study that detected grey matter intensity increase in the caudate and cerebellum bilaterally, combined with a decrease in the putamen and thalamus bilaterally [11]. In a cross sectional study between BEB and healthy controls, grey matter increases were seen in the right middle frontal gyrus, while lower grey matter volume was detected in the left post central and left superior temporal gyrus [12]. Functional cross sectional studies on BEB and cervical dystonia have shown increased basal ganglia activation with motor tasks [13]. However, these functional and structural changes demonstrated in patients with focal or generalized dystonia in comparison to healthy controls cannot be clearly classified as primary or secondary. Similarly there is no clear data on cerebral morphological changes in patients with HFS. The continuous muscular activity might directly or indirectly modify the cortical sensory-motor network, leading to secondary structural changes. To improve our understanding of dystonic conditions an exact characterization of primary structural integrity changes are necessary. Continuous tonic or phasic movements induce at short intervals secondary functional and structural alterations of the involved cerebral networks that can be then barely distinguished from elemental pathophysiological fingerprints of the disease. The purpose of our study was to analyze the cortical thickness in BEB compared to HFS patients. Both conditions present with clinically similar symptoms, but exhibit different pathophysiological backgrounds. In the proposed analysis of both groups a clear differentiation of primary and secondary cause of these morphological changes can be estimated. Moreover we postulate that the primary and secondary structurally modified networks respond to the changed sensorimotor input with immobilization after BoNT in a different way. Therefore the selected group of HFS patients was recruited as controls with a very similar clinical presentation for BEB patients. We contrast BEB and HFS patients with healthy controls in order to improve comparability and track the primary and secondary functional and morphometric changes by chronic muscular hyperactivity.

## Materials and Methods

### Patients

13 patients with BEB and 11 patients with HFS were included in this study. The demographic details are given in Table 1. Participants were studied twice: 1. at the time point of maximal symptomatic phase and 4 weeks later. A clinical examination including Unified Dystonia Rating Scale (UDRS), Blepharospasm Rating Scale (BRS),[14], Blepharospasm Disability Scale (BDS) and the Severity Rating Scale (SRS) was performed [15]. During the first session each patient was treated with intramuscular BoNT. The intramuscular injection was performed by clinicians with expertise and specialized training in BoNT administration, who were blinded to the aim of this study. BEB and HFS diagnosis was based on previously published criteria[16]. Additionally, as a control experiment we did a cross sectional analyses between the two patient groups at time 1 and 20 age and sex matched healthy controls. The demographics of the healthy controls are given in Table 1.

**Table 1:** Demographic description of groups.

	BEB	HFS	HC	*P*_*1*_	*P*_*2*_	*P*_*3*_
**Age**	65.04±6.21	58.62±11.18	57.52±9.11	0.089	0.074	0.86
**Gender**	4/9	4/7	10/10	0.92	0.24	0.12
**BoNT_ R, U**	37.69±27.20	32.73±37.37	-	0.71	-	-
**BoNT_L, U**	36.92±27.58	25.00±36.61	-	0.37	-	-

*Abbreviations*: *HC = Healthy controls; BoNT = botulinum neurotoxinR/L = right side/ left side treatment was applied to the right/left side of face; U = units; p = p-value*, *significance of differences; P*_*1*_ *–*Comparison between BEB and HFS; *P*_*2*_ *–*Comparison between BEB and HC; *P*_*3*_ *–*Comparison between HFS and HC;

### Clinical evaluation

The UDRS comprised ratings for 14 body areas including eyes and upper face, lower face, jaw and tongue, larynx, neck, trunk, shoulder/proximal arm, distal arm/hand, proximal leg, distal leg/foot. For each of the 14 body areas assessed, the UDRS quantified a severity and a duration score. The severity scale ranged from 0 (no dystonia) to 4 (extreme dystonia). The duration scale measured whether dystonia occurred at rest or with action, and the scale ranged from 0 to 4. Both, the severity and the duration score summarized to the total UDRS score. We applied the BRS to evaluate the location, influencing factors, severity of involuntary movements and disability. Higher BRS scores indicated increased disability due to BEB. With the BDS we quantified additional impairments in everyday life, and we assessed 8 domains at a scale from 1 (uncomfortable, but no limitation) to 5 points (marked limitation due to BEB). The treatment efficacy of BoNT was rated by ‘relief’ of ‘improvement’ of symptoms without specifying the nature of the improvement. We adopted the SRS to determine the severity of BEB on a scale from 0 (no BEB present) to 4 (severe, forceful contractions). In all participants we excluded any history of neurologic or psychiatric disorders as well as antidepressive, neuroleptic or sedative medications. The clinical evaluation is summarized in Table 2. Paired samples t-tests were used for the between groups comparison. All participants gave written informed consent before the study. The study was conducted in full accordance to the Declaration of Helsinki and had been approved by the local ethics committee in Medical faculty University clinic, Kiel.

**Table 2:** Clinical scores from both groups.

	BEB	HFS
**UDRS at t1**	4.27±2.37	4.64±2.61
**UDRS at t2**	2.69±1.38	3.27±2.21
**BRS at t1**	8.85±2.38	9.82±3.66
**BRS at t2**	6.69±2.95	8.09±3.73
**BDS at t1**	10.38±2.63	7.73±0.65
**BDS at t2**	9.46±2.18	7.45±0.52
**SRS at t1**	2.23±0.83	2.09±0.70
**SRS at t2**	1.46±0.66	1.09±0.83

Abbreviations: UDRS = Unified Dystonia Rating Scale; BRS = Blepharospasm Rating Scale; BDS = Blepharospasmus Disability Scale; SRS = Severity Rating Scale; t1 = time 1; t2 = time 2;

### Image acquisition and analyses

Images were acquired at a 3 Tesla MRI scanner (Achieva; Philips, Best, the Netherlands) equipped with an 8-channel head coil. A T1-weighted echo-planar imaging sequence (1 mm slice thickness, 208 × 208 matrix, TE = 3.6 ms, TR = 7.8 ms, flip angle = 8 degrees) was used. Cortical reconstruction and volumetric segmentation was performed with FreeSurfer 5.3 image analysis suite (Massachusetts General Hospital, Harvard Medical School; http://surfer.nmr.mgh.harvard.edu). Briefly, this included motion correction and averaging of multiple volumetric T1 weighted images, removal of non-brain tissue using a hybrid surface deformation procedure[17], automated Talairach transformation, segmentation of the subcortical white matter and deep gray matter volumetric structures[18], intensity normalization[19], tessellation of the gray/white matter boundary, automated topology correction[20], and surface deformation following intensity gradients to optimally place the gray/white and gray/cerebrospinal fluid borders[21].

Images were automatically processed with the longitudinal module included in Freesurfer [22]. The exact procedure is described elsewhere [23].

For the analysis, we applied a general linear model of statistical analysis to establish the cross-sectional differences between the groups. Further we quantified the longitudinal changes by computing the rate of change of cortical thickness (mm/year) between the groups using the formula: (thickness at Time 2 –thickness at Time 1) / (Time 2 –Time 1). Cortical thickness was smoothed with a 10-mm Gaussian kernel to reduce local variations in the measurements [24]. Statistical differences were computed using a random effects model with t-tests for each cortical location. For statistical difference maps the significant threshold was set to an uncorrected p-value of ≤0.001 (two-tailed). Further, correction using the Monte-Carlo based on the simulation (cluster analyses) adjustment was applied with a *p*-value of 0.05. Finally, we estimated the Pearson correlation (corrected for multiple comparisons using Bonferroni correction) between the change in clinical scores and the change in cortical thickness for both the BEB and HFS group separately for each hemisphere.

## Results

There were no significant differences between the groups in respect to demographics, administered dosage UDRS, BRS and SRS scale (Table 2). BoNT treatment improved the dystonic contractions in BEB patients, as measured with the UDRS and SRS scales (Table 2). HFS patients improved as well at time 2 on the SRS scale. The groups differed in the level of disability (BD scale) at baseline as well as at time 2 after BoNT treatment.

We performed cross-sectional analyses between the BEB at time 1 and the healthy controls, and found that cortical thinning occurred in BEB at the left temporal pole, right lateral occipital, right superior frontal and right inferior temporal regions. Similarly, in the comparison of HFS patients to HC, we found a cortical thinning in subjects with HFS at the left lingual and left posterior bank of transversal temporal gyrus. The complete description of the results with t-values, voxel size and Talairach coordinates are given in Tables 3 and 4.

**Table 3:** Statistical significant clusters for the group comparison between patients with BEB and HC.

Right Hemisphere						
Cluster No	t-Max	Size (mm^2^)	Tal X	Tal Y	Tal Z	Annotation
**1**	-4.5331	116.32	26.7	-97.7	-3.6	Lateral Occipital
**2**	-4.0909	95.44	7.0	-0.6	64.6	Superior Frontal
**3**	-3.6567	29.55	46.6	-5.6	-33.2	Inferior Temporal
**Left Hemisphere**						
**1**	-4.0565	124.91	-34.9	5.2	-30.7	Temporal pole

**Table 4:** Statistical significant clusters for the group comparison between patients with HFS and HC.

Left Hemisphere						
**1**	-4.0565	52.14	-13.6	-54.0	-2.9	Lingual
**2**	-4.0414	21.11	-55.1	-42.6	-4.0	Posterior part of the tranverse temporal gyrus

**Fig 1:**
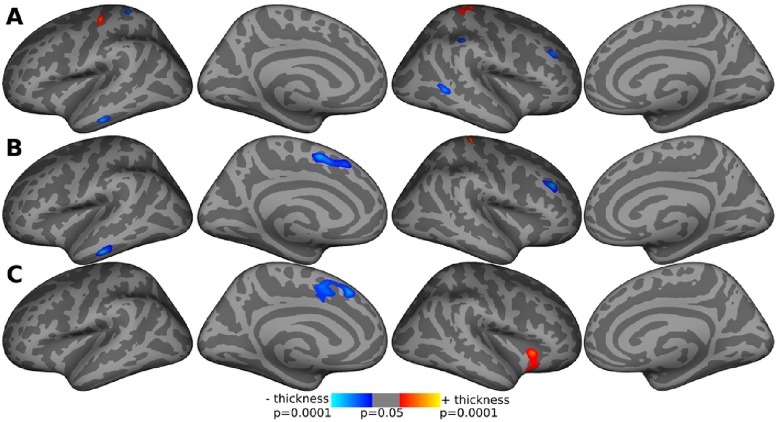
Cortical thickness differences between the BEB and HFS groups. (A) cross-sectional analysis between BEB and HFS at baseline; (B) cross-sectional analysis between BEB and HFS after the treatment; (C) longitudinal rate of change of cortical thickness over time in BEB compared to HFS. Only clusters that survived the *P<*0.001 threshold are included. Images are presented at *P* = 0.05 to better show the extent of cortical changes.

The analysis of cortical thickness has been performed in two stages. First, we performed an intergroup cross-sectional analysis at time 1, i.e. BEB vs. HFS at time 1 and an additional analysis at time 2 (Fig 1). In comparison to HFS, the BEB group at time 1 revealed differences in cortical thickness in the primary motor cortex bilaterally, left inferior temporal, right middle temporal, right frontal-rostral and right supramarginal regions. After BoNT-treatment, cortical thickness changed in the primary motor cortex and in the left pre-SMA of the BEB group compared to HFS.

The analysis for each group individually revealed no cortical thickness changes over time. The differential analysis of the longitudinal change between the groups showed a higher rate of cortical thinning in BEB patients over time in the left pre-SMA and the right insula in comparison to HFS subjects. From the correlation analyses of change in clinical parameters to the change in cortical thickness we found the change in cortical thickness in BSF patients over time (t0-t1) was significantly (p = 0.0105) correlated (r = -0.6804) to change (t0-t1) in BRS only for the right hemisphere. No other significant correlations were found for any other clinical parameters or for the HFS group.

## Discussion

Patients with BEB exhibited bilateral increased cortical thickness in the sensorimotor cortex compared to HFS. Cortical thickness decreased in BEB patients after BoNT treatment while no changes were noted in the HFS group. This finding might be attributed to cortical reorganization over time after BoNT treatment. The exact functional and structural changes that occur with improvement of symptoms in patients with dystonic or hyperkinetic disorders after BoNT therapy are not clear. Previous studies demonstrated that long term potentiation (LTP)–like plasticity studied using transcranial magnetic stimulation was abnormal in BEB patients, but could be restored after BoNT treatment [25]. Possibly these functional changes are reflected in our study by the decreased cortical thickness in the sensorimotor cortex after the BoNT in BEB patients. Systematically the somatotopic representations of punctate tactile stimuli were mapped before and after BoNT therapy and showed deficient activation in primary and secondary somatosensory representations and described modulation of basal ganglia activation might reflect an indirect effect of the BoNT [26]. The longitudinal structural adaptation in our study was specific for BEB patients pointing out a disease specific reorganization. The morphological differences involved the motor system, but were also accompanied by changes in the secondary and tertiary associative cortices.

Previous studies suggested that BEB pathophysiology is associated with changes in the primary motor cortex [27]. Specifically, gray matter reductions as shown by voxel-based morphometry (VBM) in the facial portion of the primary motor cortex have been described in BEB when compared to healthy controls [28]. This result was not replicated by our data. The significant cluster in the comparison of BEB patients with HC was more rostral and could be related to M1 interconnected areas in the frontal cortex. For the main hypothesis of our study we address however the contrast of BEB and HFS patients to exclude unspecific and secondary changes. A further great advantage of our analysis is the use of cortical thickness measurements and not VBM-related intensity analysis, which might represent a less sensitive measure of grey matter integrity. Therefore, our analysis showed higher cortical thickness values in the motor cortex of BEB patients. Dynamic and symptom-specific structural changes that involve the primary motor cortex might be an important fingerprint of BEB in comparison to HFS but also healthy controls, as shown previously. Structural alterations in the primary motor cortex have been demonstrated in patients with writer’s cramp dystonia by our group [29], and in spasmodic dysphonia [30].

Our current analysis underlines further the primary role of pre-SMA in the BEB pathophysiology in comparison to HFS subjects. In this region, the rate of cortical thinning was also much higher and to a greater extent after BoNT than in the HFS group. Pre-SMA is directly involved in the control of involuntary actions [31] and shows over-activity during dyskinesia’s in PD patients [32]. Direct electrical stimulation of pre-SMA can elicit an ‘urge’ to move a specific body part [33]. Neurons in the pre-SMA have been shown to respond during change-of-plan, altering movement plans [34] and during learning activity for a complex sequence of hand movements [35]. Hence, structural pre-SMA changes in BEB might be associated with an altered process of involuntary movement initiation and adaptation to a new order of movements [36]. Thus, its direct structural involvement in the studied patient group is in line with the functional role of this region. The comparison to the healthy controls did not reveal any significant changes in the primary motor cortex or pre-SMA indicating these regions is specific to the structural changes between BEB and HFS patients. First limitation of the study on the interpretation of the results between the patients and the HC is there is no test-retest reliability in this study. Second limitation of the study is a higher variance and structural changes at the time point of maximal symptoms manifestation may preclude detection of treatment effects in each of the corresponding groups.

After BoNT treatment and with symptomatic improvement, BEB patients showed cortical thinning in the left inferior temporal, left pre-SMA, right frontal-rostral and primary motor area. No clear changes of cortical thickness were found in the HFS group after BoNT treatment. The clinical effects of treatment are similar in both groups, namely a decreased rate of spontaneous muscular contractions in facial muscles, but nevertheless, different structural changes were found to occur in the analyzed groups. We hypothesize that the achieved immobilization and improvement of the hyperkinetic movement disorder has different effects on cortical plasticity in the two groups: in BEB a normalization of restructured information flow through cortico-cortical and basal-ganglia-cortical circuits might occur through immobilization which in turn might modify cortical integrity; in HFS BoNT therapy manifests could merely as a symptomatic effect.

## Conclusion

This study shows that standard BoNT therapy brings both clinical improvements as well as significant cortical and subcortical reorganization patterns. We hypothesize that BoNT-associated immobilization of the affected muscles might modify the input in the cortical circuit and cortico-basal-ganglia loops, which could result in a cortical structural reorganization in order to adapt to new motoric stimuli.
