# Correction: Grey Matter Microstructural Integrity Alterations in Blepharospasm Are Partially Reversed by Botulinum Neurotoxin Therapy

**DOI:** 10.1371/journal.pone.0172374

**Published:** 2017-02-13

**Authors:** Alexandru Hanganu, Muthuraman Muthuraman, Venkata Chaitanya Chirumamilla, Nabin Koirala, Burcu Paktas, Günther Deuschl, Kirsten E. Zeuner, Sergiu Groppa

The first author’s name appears incorrectly in the byline and the citation. The correct name is: Alexandru Hanganu. The correct citation is: Hanganu A, Muthuraman M, Chirumamilla VC, Koirala N, Paktas B, Deuschl G, et al. (2016) Grey Matter Microstructural Integrity Alterations in Blepharospasm Are Partially Reversed by Botulinum Neurotoxin Therapy. PLoS ONE 11(12): e0168652. doi:10.1371/journal.pone.0168652
